# Rapid Detection of the mt3243A > G Mutation Using Urine Sediment in Elderly Chinese Type 2 Diabetic Patients

**DOI:** 10.1155/2017/4683857

**Published:** 2017-06-21

**Authors:** Yinan Zhang, Xiujuan Du, Xinqian Geng, Chen Chu, Huijuan Lu, Yixie Shen, Ruihua Chen, Pingyan Fang, Yanmei Feng, Xiaojie Zhang, Yan Chen, Yanping Zhou, Congrong Wang, Weiping Jia

**Affiliations:** ^1^The Metabolic Diseases Biobank, Center for Translational Medicine, Shanghai Key Laboratory of Diabetes, Shanghai Jiao Tong University Affiliated Sixth People's Hospital, Shanghai 200233, China; ^2^Department of Endocrinology and Metabolism, Shanghai Key Laboratory of Diabetes, The Metabolic Diseases Biobank, Shanghai Jiao Tong University Affiliated Sixth People's Hospital, Shanghai 200233, China; ^3^Department of Otolaryngology Head and Neck Surgery, Shanghai Jiao Tong University Affiliated Sixth People's Hospital, Shanghai 200233, China; ^4^Department of Neurology, Shanghai Jiao Tong University Affiliated Sixth People's Hospital, Shanghai 200233, China; ^5^Department of Ophthalmology, Shanghai Jiao Tong University Affiliated Sixth People's Hospital, Shanghai 200233, China; ^6^Department of Ophthalmology, Shanghai First People's Hospital Affiliated to Shanghai JiaoTong University School of Medicine, Shanghai 200080, China

## Abstract

**Objective:**

In this study, we aimed to identify mt3243A > G mutation carriers in a group of Chinese elderly type 2 diabetic patients by a rapid and noninvasive diagnostic system.

**Methods:**

DNA was extracted from blood, saliva, and urine sediment samples. The mutation screening and quantitation of heteroplasmy were performed by high-resolution melting (HRM) curve and pyrosequencing, respectively. Patients with mt3243A > G mutation underwent a detailed audiometric, ophthalmologic, neurological, and cardiac examination.

**Results:**

Two patients (2/1041) carrying the mt3243A > G mutation were detected among all type 2 diabetic patients. In patient 1, the heteroplasmy was 0.8%, 2.8%, and 14.7% in peripheral blood leukocytes, saliva, and urine sediment, respectively. In patient 2, the heteroplasmy was 5.3%, 8.4%, and 37.7% in peripheral blood leukocytes, saliva, and urine sediment, respectively. Both of the two patients showed hearing impairment. Abnormal ophthalmologic conditions and hyperintensity on T2-weighted magnetic resonance images were showed in patient 1.

**Conclusion:**

The occurrence of mt3243 A > G mutation was 0.2% in Chinese elderly type 2 diabetic patients. Moreover, detection of mt3243 A > G mutation in urine sediment with high-resolution melting (HRM) curve and pyrosequencing is feasible in molecular genetic diagnosis.

## 1. Introduction

Mitochondrial diabetes, which is known as a maternally transmitted clinical subtype of diabetes, is most commonly caused by an A to G substitution in the mitochondrial DNA at position 3243 (mt3243A > G mutation), which encodes tRNALeu(UUR) [1]. Since it was first identified in 1990, the mt3243A > G mutation has been well investigated in many ethnic groups [2–6]. The reported prevalence of the mt3243A > G mutation varies from study to study, largely due to different races of patients and the selection criteria [7–11]. The prevalence of the mt3243A > G mutation among patients with type 2 diabetes (T2DM) in China and Finland is 0.4% and 1.0%, respectively [8, 12]. This prevalence increases to 5.9% in early onset patients with a family history of diabetes and reaches 60% in patients with both T2DM and deafness [5, 11].

On the other hand, the clinical phenotype of the mt3243A > G mutation is variable, partially due to the number of affected mitochondria in different tissues. This characteristic is referred to as heteroplasmy. Levels of heteroplasmy can vary between tissues and even within one tissue in a single patient over time [1]. In previous studies, DNA extracted from peripheral blood samples was screened using PCR restriction fragment length polymorphism (PCR-RFLP) analysis and Sanger sequencing [1]. However, other studies, including our own, have demonstrated that heteroplasmy is lower in peripheral blood than in urine sediment, suggesting that the detection rate could be improved by using urine sediment [13–16]. In addition, our previous study also showed that high-resolution melting (HRM) curve analysis is a rapid and sensitive method to screen for this mutation in urine sediment. Samples from mt3243A > G mutation carriers were differentiated by distinct melting curves in HRM, and subsequent pyrosequencing validated positive samples and quantified the level of heteroplasmy. In contrast, the level of heteroplasmy could not be quantified by Sanger sequencing, as the peak height was not proportional to the degree of heteroplasmy [16]. Therefore, the implementation of a noninvasive diagnostic method to detect the mt3243A > G mutation in urine sediment by HRM analysis combined with pyrosequencing is expected to detect more patients carrying the mt3243A > G mutation.

In this study, we aimed to identify mt3243A > G mutation carriers in urine sediment samples from a group of elderly Chinese type 2 diabetic patients.

## 2. Materials and Methods

### 2.1. Subjects

Data were obtained from a community-based peer-education project conducted between January 2013 and December 2015. A total of 1041 patients with T2DM who completed a baseline questionnaire were enrolled in this study. The mean age at recruitment and age at diagnosis of the cohort was 67.2 ± 6.7 years and 54.8 ± 8.1 years, respectively. Body mass index (BMI) was calculated as weight in kilograms divided by height in metres squared. The diagnosis of diabetes was confirmed according to the 1999 World Health Organization (WHO) criteria [17]. The study was approved by the Institutional Review Board of Shanghai Jiao Tong University Affiliated Sixth People's Hospital and conducted in accordance with the principles of the Declaration of Helsinki. Written informed consent was obtained from each participant.

### 2.2. DNA Samples

Urine sediment samples were collected from 1041 patients. Blood, saliva, and urine sediment samples were collected from patients who were identified as carrying the mt3243A > G mutation and from their family members. DNA was extracted and purified using an automated nucleic acid extraction instrument (Lab-Aid 820; BioV, China). The eluted DNA concentration was measured using a NanoDrop spectrophotometer, and the DNA was diluted to 10 ng/μl.

### 2.3. High Resolution Melting Curve (HRM) Analysis

PCR reactions were performed in a Rapid Cycler (Idaho Technology, Salt Lake City, UT) using the primers MT3243-F (5′-TTCACAAAGCGCCTTCCCCC-3′) and MT3243-R (5′-CCATTGCGATTAGAATGGGTACA-3′). Screening of the mt3243A > G mutation was undertaken on PCR products using HRM analysis (Idaho Technology). Melting was conducted from 75°C to 95°C at 0.3°C per second. The melting curves were analysed by the software, with normalized curves for comparison among samples.

### 2.4. Pyrosequencing

PCR products from patients carrying the mt3243A > G mutation detected by HRM analysis were analysed using pyrosequencing according to the previous report [16].

### 2.5. Clinical Evaluation of Patients with the mt3243A > G Mutation

Information about patients with the mt3243A > G mutation was obtained by a standard questionnaire and a face-to-face interview. Laboratory examinations included routine urine and blood tests, HbA1c, fasting plasma glucose, 2 h postprandial plasma glucose, fasting C-peptide, 2 h postprandial C-peptide, glutamic acid decarboxylase (GAD-Ab), islet antigen 2 (IA2-Ab), cardiac enzyme, lipid profile, liver and renal biochemistry profile, and electrolyte profile.

Audiometric, ophthalmologic, neurological, and cardiac examinations were performed to assess the effect of the mt3243 A > G mutation on multiorgan involvement. Diagnosis of hearing loss was based on audiometric measurements, including air-conduction pure-tone thresholds, bone-conduction thresholds, tympanometry, and distortion-product otoacoustic emission (DPOAE). A standardized ophthalmologic examination was performed including best-corrected visual acuity of both eyes, ophthalmoscopy after pupillary dilation, fundus photograph electrooculogram (EOG), flash electroretinogram (FERG), multifocal ERG (mfERG), and optical coherence tomography scans (OCT) to give insight into the current functional status of the patient. To evaluate neurological function, a neurologic physical examination, electromyography (EMG), electroencephalogram (EEG), and brain magnetic resonance imaging (MRI) were performed. To evaluate cardiac function, electrocardiogram (EKG) and echocardiography were conducted.

### 2.6. Statistical Methods

Characteristics of participants were presented by descriptive statistics. Data were analysed using SPSS version 21.0 (SPSS Inc., Chicago, IL) and expressed as the mean ± SD for continuous variables.

## 3. Results

### 3.1. Screening for Mutation in the Studied Population

The clinical characteristics of patients with T2DM are summarized in Table 1. By comparing the melting curves of patients to healthy controls, two patients (2/1041) carrying the mt3243A > G mutation were detected among all patients with T2DM. In addition, quantification of the heteroplasmy of the mutation in the two patients was further analysed by pyrosequencing with DNA from peripheral blood leukocytes, saliva, and urine sediment samples. The measured heteroplasmy was adjusted by a cubic polynomial curve according to our previous study [16]. In patient 1, the heteroplasmy was 0.8%, 2.8%, and 14.7% in peripheral blood leukocytes, saliva, and urine sediment, respectively. In patient 2, the heteroplasmy was 5.3%, 8.4%, and 37.7% in peripheral blood leukocytes, saliva, and urine sediment, respectively.

### 3.2. Clinical Case Presentation

Patient 1 (F1955II-1) was a 74-year-old man who had been diagnosed with T2DM at the age of 56 (Table 2). He had been treated with metformin for 18 years until he was identified as carrying the mt3243A > G mutation in this study. His BMI was 23.7 kg/m^2^. He had lower levels of LDL-c and fasting C-peptide. The patient had never undergone any episodes of hypoglycaemia, diabetic ketoacidosis, or lactic acidosis.

**Figure 1:**
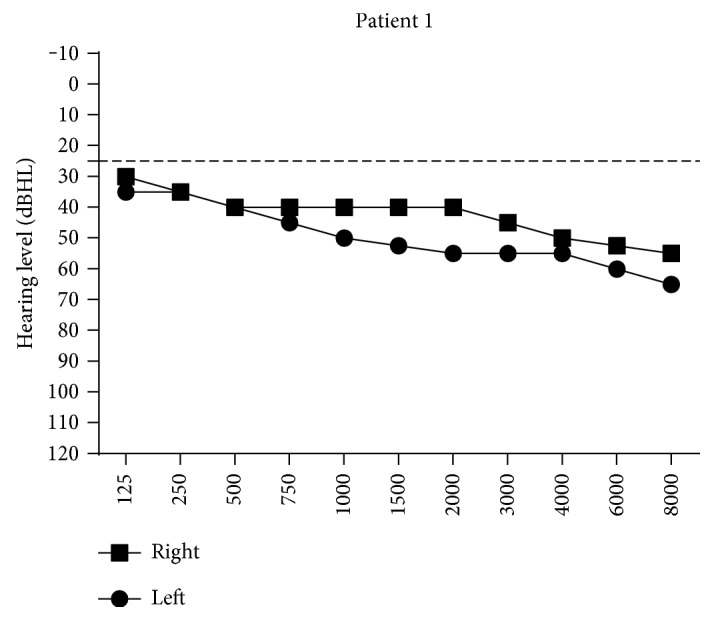
Bilateral pure-tone audiometry in patient 1. An ear hearing level equals or less than 25 dB was considered normal.

**Figure 2:**
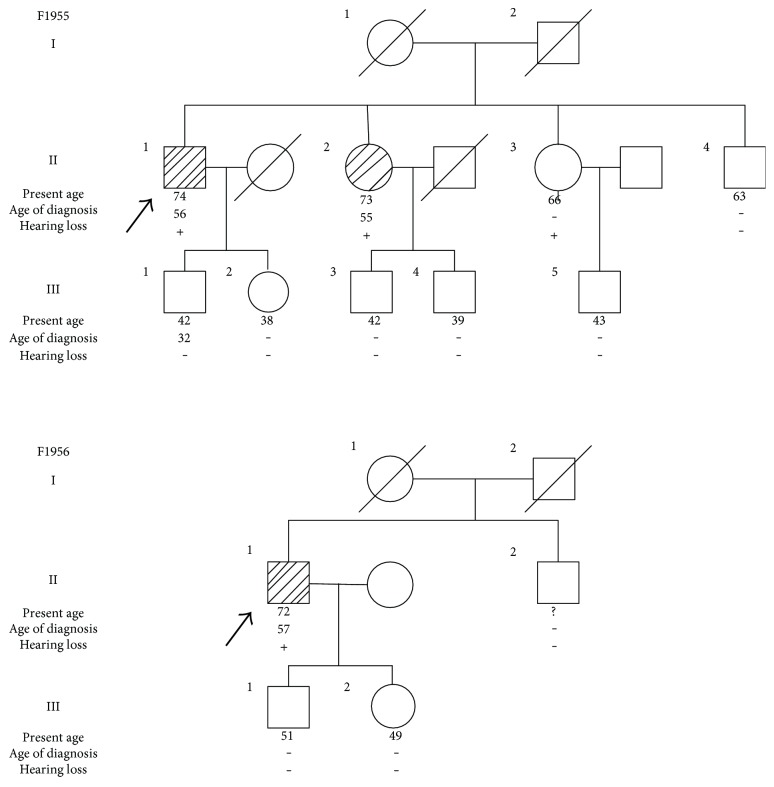
Pedigree of two families with mt3243A > G mutation (F1955 and F1956). ○ = female; □ = male; 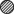, ▨ = patients with mt3243A > G mutation; Ø, 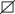 = deceased. The arrow indicates the probands. Present age, age of diagnosis, and hearing loss are also shown.

In this study, the patient had newly diagnosed bilateral sensorineural hearing loss at all frequencies according to the results of the audiogram (Figure 1). Best-corrected visual acuity (BCVA) was 20/25 bilateral. Fundus photograph showed no obvious diabetic retinopathy. Optical coherence tomography (OCT) image of the right eye was normal; however, a drusen-like hyperreflective lesion was found in the left eye on OCT image. This dome-shaped change was likely to originate from the retinal pigment epithelium (RPE), which induced IS/OS disruption. In addition, ophthalmic electrophysiology was performed to detect the visual function. Bilateral eyes had normal function of electrooculogram (EOG), while flash electroretinogram (FERG) suggested a decrease of b wave in bright adaption. Furthermore, multifocal ERG (mfERG) showed a decrease of amplitudes bilateral. Hypomnesia was present from the age of 56. The main findings of the MRI were hypointensity on T1-weighted images and hyperintensity on T2-weighted images of the bilateral frontal lobe, bilateral parietal lobe, basal ganglia and corona radiata, indicating cerebral ischaemia, lacunar infarction, and cerebral atrophy. Echocardiography showed tricuspid and aortic regurgitation, though the patient's blood pressure was normal. One of his siblings (F1955II-2) was also found to carry the mt3243A > G mutation and presented with hearing loss without being exposed to noise. Patient F1955II-2 was diagnosed with diabetes at the age of 55. The patient's parents were deceased, and their medical history of diabetes and the mt3243A > G mutation were not available (Figure 2).

Patient 2 (F1956II-1) was a 72-year-old man diagnosed with T2DM at the age of 57 (Table 2). Clinical examination results, including audiogram, were not available as a result of the patient's severe lower limb fractures. He had been treated with metformin for 15 years until he was identified as carrying the mt3243A > G mutation in this study. He had higher levels of HbA1c (7.9%) and 2 h postprandial plasma glucose level (12.40 mmol/L). His BMI was 19.2 kg/m^2^. Self-reported hearing loss was present since the age of 50 without being exposed to noise, and the patient reported the development of blurred vision at the age of 62. Genetic testing of his family members revealed that none of them carried the mt3243A > G mutation. The patient's parents were deceased, and their medical histories of diabetes and the mt3243A > G mutation were not available (Figure 2).

## 4. Discussion

In this study, we screened for the mt3243A > G mutation in urine sediments of elderly Chinese T2DM patients using HRM analysis combined with pyrosequencing. We found that the occurrence of the mt3243A > G mutation was 0.2% (2 of 1041 patients) in elderly Chinese T2DM patients. The occurrence of this mutation (0.2%) in elderly T2DM patients (ages ranging from 47 to 85 years) was similar to that observed in unrelated non-insulin-dependent diabetes mellitus (NIDDM) patients (0.3%, ages ranging from 8 to 58 years) [5]. Because metformin can increase the risk of lactate acidosis in T2DM patients with the mt3243A > G mutation, it is worthwhile screening for this mutation before treatment despite its low occurrence in elderly T2DM patients.

The most common genetic methods for the detection and quantitation of the mt3243A > G mutation in previous studies were PCR-RFLP and Sanger sequencing, and peripheral blood was the most common source of material for genetic screening. However, the level of heteroplasmy in peripheral blood is much lower than that in urine sediment and tends to decline over time [13–16]. Thus, urine sediment is an ideal choice of material for screening for the mt3243A > G mutation in elderly patients. On the other hand, PCR-RFLP analysis and Sanger sequencing are often used to detect the mt3243 A > G mutation. However, Yan et al. reported that PCR-RFLP and Sanger sequencing only detect the mt3243A > G mutation when the level of heteroplasmy is more than 5–10%, while the sensitivity of HRM (2%) is much higher [16, 18]. Moreover, pyrosequencing can accurately quantify the level of heteroplasmy from 0% to 100% [16]. Our study confirms the feasibility of screening for the mt3243 A > G mutation in urine sediment by HRM followed by pyrosequencing in an elderly T2DM cohort. Consistent with previous studies, the results of this study showed that the heteroplasmy of both patients carrying the mt3243 A > G mutation increased from peripheral blood leukocytes to urine sediment [13, 14].

It is well known that patients with the mt3243 A > G mutation have a wide variety of disease manifestations. In this study, audiometric, ophthalmologic, neurological, and cardiac examinations were performed to assess the effects of the mt3243A > G mutation on multiple organs. Both of the two patients showed hearing loss. Patient 1 had characteristic bilateral sensorineural hearing loss at all frequencies and patient 2 showed self-reported hearing loss, which is consistent with previous studies showing that approximately more than half of diabetic patients carrying this mutation have sensorineural hearing loss [19]. For the eyes, mfERG indicated foveal photoreceptor dysfunction bilaterally while RPE function had no obvious abnormality from EOG examination. However, OCT scanning showed a drusen-like hyperreflective lesion in the left eye. This dome-shaped change was likely to originate from RPE. These findings imply that foveal photoreceptors might have dysfunction accompanied with or without RPE abnormality in mt3243 A > G mutation carriers as previously reported [20, 21]. Even in the absence of stroke-like episodes, MRI of patient 1 showed widespread hyperintensity on T2-weighted images of the bilateral frontal lobe, bilateral parietal lobe, basal ganglia, and corona radiata, indicating cerebral ischaemia, lacunar infarction, and cerebral atrophy. Interestingly, previous studies also reported hyperintensity of the bilateral basal ganglia and corona radiata on T2-weighted images in mt3243A > G mutation carriers, either with or without mitochondrial encephalopathy, lactic acidosis, and stroke-like episodes (MELAS) syndrome [22–24]. Further research is warranted to investigate the relationship between T2-weighted images and the mt3243A > G mutation.

In conclusion, our study found that the occurrence of the mt3243A > G mutation was 0.2% in elderly Chinese T2DM patients. Furthermore, screening for the mt3243A > G mutation in urine sediment using HRM and pyrosequencing can make a rapid and accurate etiological diagnosis possible and thus contributes to precision and personalized medicine.

## Figures and Tables

**Table 1:** The clinical characteristics of patients with type 2 diabetes.

Clinical characteristics	Diabetic subjects (*n* = 1041)
Age	67.2 ± 6.7
Age of diagnosis (years)	54.8 ± 8.1
Male (%)	36.2
BMI (kg/m^2^)	26.3 ± 5.0
HbA1c (%)	7.2 ± 1.3

BMI denotes body mass index.

**Table 2:** The biochemical and clinical characteristics of two patients with T2DM carrying the mt3243 A > G mutation.

Clinical and biochemical characteristics	Patient 1 (F1955II-1)	Patient 2 (F1956II-1)
Age (years)	74	72
Age of diagnosis (years)	56	57
Sex (M/F)	M	M
BMI (kg/m^2^)	23.7	19.5
Blood pressure (mmHg)		
SBP	124	108
DBP	68	68
HbA1c (%)	5.9	7.9
Fasting plasma glucose (mmol/L)	5.24	6.92
2 h postprandial plasma glucose (mmol/L)	10.25	12.40
Fasting C-peptide (ng/mL)	0.64	1.21
2 h postprandial C-peptide (ng/mL)	2.30	2.89
GAD-Ab (U/mL)	0.00	0.00
IA2-Ab (U/mL)	0.00	0.00
Triglyceride (mmol/L)	0.61	1.09
Total cholesterol (mmol/L)	3.48	4.11
HDL-c (mmol/L)	1.65	0.99
LDL-c (mmol/L)	1.61	2.50
Urine ketones	Negative	Negative
ACR (μg/mg)	9.23	152.55
Creatinine kinase (U/L)	104	22
Treatment	Oral drugs, insulin	Oral drugs
Thyroid disease	No	No
Self-reported hearing impairment	No	Yes
Audiogram	Bilateral sensorineural hearing loss	NA
Neurological involvement	Hypomnesia	No
MRI	Hypointensity on T1-weighted images and hyperintensity on T2-weighted images of the bilateral frontal lobe, bilateral parietal lobe, basal ganglia, and corona radiata	NA
Retinal OCT	IS/OS disruption	NA

BMI: body mass index; HDL-c: high-density lipoprotein cholesterol; LDL-c: low-density lipoprotein cholesterol; ACR: urinary albumin to creatinine ratio; GAD-Ab: glutamic acid decarboxylase (GAD-Ab); IA2-Ab: islet antigen 2 (IA2-Ab); NA: not available; OCT: optical coherence tomography; IS/OS: inner/outer segment.

## References

[1] Murphy R., Turnbull D. M., Walker M., Hattersley A. T. (2008). Clinical features, diagnosis and management of maternally inherited diabetes and deafness (MIDD) associated with the 3243A>G mitochondrial point mutation. *Diabetic Medicine : A Journal of the British Diabetic Association*.

[2] Goto Y., Nonaka I., Horai S. (1990). A mutation in the tRNA(Leu)(UUR) gene associated with the MELAS subgroup of mitochondrial encephalomyopathies. *Nature*.

[3] Reardon W., Ross R. J., Sweeney M. G. (1992). Diabetes mellitus associated with a pathogenic point mutation in mitochondrial DNA. *Lancet (London, England)*.

[4] Verma A., Moraes C. T., Shebert R. T., Bradley W. G. (1996). A MERRF/PEO overlap syndrome associated with the mitochondrial DNA 3243 mutation. *Neurology*.

[5] Xiang K., Wang Y., Wu S. (1997). Mitochondrial tRNA(Leu(UUR)) gene mutation diabetes mellitus in Chinese. *Chinese Medical Journal*.

[6] Katulanda P., Groves C. J., Barrett A. (2008). Prevalence and clinical characteristics of maternally inherited diabetes and deafness caused by the mt3243A > G mutation in young adult diabetic subjects in Sri Lanka. *Diabetic Medicine : A Journal of the British Diabetic Association*.

[7] Saker P. J., Hattersley A. T., Barrow B. (1997). UKPDS 21: low prevalence of the mitochondrial transfer RNA gene (tRNA(Leu(UUR))) mutation at position 3243bp in UK Caucasian type 2 diabetic patients. *Diabetic Medicine : A Journal of the British Diabetic Association*.

[8] Ji L., Hou X., Han X. (2001). Prevalence and clinical characteristics of mitochondrial tRNAleu(UUR) nt 3243 A-->G and nt 3316 G-->A mutations in Chinese patients with type 2 diabetes. *Diabetes Research and Clinical Practice*.

[9] Holmes-Walker D. J., Mitchell P., Boyages S. C. (1998). Does mitochondrial genome mutation in subjects with maternally inherited diabetes and deafness decrease severity of diabetic retinopathy?. *Diabetic Medicine : A Journal of the British Diabetic Association*.

[10] Vionnet N., Passa P., Froguel P. (1993). Prevalence of mitochondrial gene mutations in families with diabetes mellitus. *Lancet (London, England)*.

[11] Kadowaki T., Kadowaki H., Mori Y. (1994). A subtype of diabetes mellitus associated with a mutation of mitochondrial DNA. *The New England Journal of Medicine*.

[12] Martikainen M. H., Ronnemaa T., Majamaa K. (2013). Prevalence of mitochondrial diabetes in southwestern Finland: a molecular epidemiological study. *Acta Diabetologica*.

[13] Shanske S., Pancrudo J., Kaufmann P. (2004). Varying loads of the mitochondrial DNA A3243G mutation in different tissues: implications for diagnosis. *American Journal of Medical Genetics. Part a*.

[14] Narbonne H., Perucca-Lostanlen D., Desnuelle C., Vialettes B., Saunieres A., Paquis-Flucklinger V. (2001). Searching for A3243G mitochondrial DNA mutation in buccal mucosa in order to improve the screening of patients with mitochondrial diabetes. *European Journal of Endocrinology*.

[15] McDonnell M. T., Schaefer A. M., Blakely E. L. (2004). Noninvasive diagnosis of the 3243A > G mitochondrial DNA mutation using urinary epithelial cells. *European Journal of Human Genetics : EJHG*.

[16] Yan J. B., Zhang R., Xiong C. (2014). Pyrosequencing is an accurate and reliable method for the analysis of heteroplasmy of the A3243G mutation in patients with mitochondrial diabetes. *The Journal of Molecular Diagnostics*.

[17] Alberti K. G., Zimmet P. Z. (1998). Definition, diagnosis and classification of diabetes mellitus and its complications. Part 1: diagnosis and classification of diabetes mellitus provisional report of a WHO consultation. *Diabetic Medicine : A Journal of the British Diabetic Association*.

[18] Smith M. L., Hua X. Y., Marsden D. L. (1997). Diabetes and mitochondrial encephalomyopathy with lactic acidosis and stroke-like episodes (MELAS): radiolabeled polymerase chain reaction is necessary for accurate detection of low percentages of mutation. *The Journal of Clinical Endocrinology and Metabolism*.

[19] Suzuki S., Hinokio Y., Hirai S. (1994). Pancreatic beta-cell secretory defect associated with mitochondrial point mutation of the tRNA(LEU(UUR)) gene: a study in seven families with mitochondrial encephalomyopathy, lactic acidosis and stroke-like episodes (MELAS). *Diabetologia*.

[20] Harrison T. J., Boles R. G., Johnson D. R., LeBlond C., Wong L. J. (1997). Macular pattern retinal dystrophy, adult-onset diabetes, and deafness: a family study of A3243G mitochondrial heteroplasmy. *American Journal of Ophthalmology*.

[21] Massin P., Virally-Monod M., Vialettes B. (1999). Prevalence of macular pattern dystrophy in maternally inherited diabetes and deafness. *GEDIAM Group. Ophthalmology*.

[22] Lien L. M., Lee H. C., Wang K. L., Chiu J. C., Chiu H. C., Wei Y. H. (2001). Involvement of nervous system in maternally inherited diabetes and deafness (MIDD) with the A3243G mutation of mitochondrial DNA. *Acta Neurologica Scandinavica*.

[23] Bowen J., Richards T., Maravilla K. (1998). MR imaging and proton MR spectroscopy in A-to-G substitution at nucleotide position 3243 of leucine transfer RNA. *AJNR. American Journal of Neuroradiology*.

[24] Vandana V. P., Bindu P. S., Sonam K. (2016). Audiological manifestations in mitochondrial encephalomyopathy lactic acidosis and stroke like episodes (MELAS) syndrome. *Clinical Neurology and Neurosurgery*.

